# Crohn’s and Colitis Canada’s 2021 Impact of COVID-19 and Inflammatory Bowel Disease in Canada: Executive Summary

**DOI:** 10.1093/jcag/gwab027

**Published:** 2021-11-05

**Authors:** M Ellen Kuenzig, Joseph W Windsor, Lisa Barrett, Charles N Bernstein, Alain Bitton, Matthew W Carroll, Usha Chauhan, Stephanie Coward, Sharyle Fowler, Jean-Eric Ghia, Rose Geist, Deanna L Gibson, Lesley A Graff, Anne M Griffiths, James Guoxian Huang, Jennifer L Jones, Reena Khanna, Peter L Lakatos, Kate Lee, David R Mack, John K Marshall, Mariam S Mukhtar, Sanjay K Murthy, Geoffrey C Nguyen, Remo Panaccione, Cynthia H Seow, Harminder Singh, Parul Tandon, Laura E Targownik, Sandra Zelinsky, Eric I Benchimol, Gilaad G Kaplan

**Affiliations:** 1 SickKids Inflammatory Bowel Disease Centre, Division of Gastroenterology, Hepatology and Nutrition, The Hospital for Sick Children, Toronto, Ontario, Canada; 2 Child Health Evaluative Sciences, SickKids Research Institute, Toronto, Ontario, Canada; 3 Department of Medicine, University of Calgary, Calgary, Alberta, Canada; 4 Department of Medicine, Dalhousie University, Halifax, Nova Scotia, Canada; 5 Department of Internal Medicine, Max Rady College of Medicine, Rady Faculty of Health Sciences, University of Manitoba, Winnipeg, Manitoba, Canada; 6 University of Manitoba IBD Clinical and Research Centre, Winnipeg, Manitoba, Canada; 7 Department of Medicine, McGill University Health Centre, McGill University, Montreal, Quebec, Canada; 8 Department of Pediatrics, University of Alberta, Edmonton, Alberta, Canada; 9 Hamilton Health Science, Department of Medicine and Farncombe Family Digestive Health Research Institute, McMaster University, Hamilton, Ontario, Canada; 10 Departments of Community Health Sciences, University of Calgary, Calgary, Alberta, Canada; 11 Department of Medicine, University of Saskatchewan, Saskatoon, Saskatchewan, Canada; 12 Department of Immunology and Internal Medicine section of Gastroenterology, Max Rady College of Medicine, Rady Faculty of Health Sciences, University of Manitoba and University of Manitoba Inflammatory Bowel Disease Clinical and Research Centre, Winnipeg, Manitoba, Canada; 13 Department of Psychiatry, University of Toronto, Toronto, Ontario, Canada; 14 Department of Biology, Faculty of Science; Department of Medicine, Faculty of Medicine, The University of British Columbia, Okanagan campus, Kelowna, British Columbia, Canada; 15 Department of Clinical Health Psychology, Max Rady College of Medicine, Rady Faculty of Health Sciences, University of Manitoba, Winnipeg, Manitoba, Canada; 16 ICES, Toronto, Ontario, Canada; 17 Department of Paediatrics and Institute of Health Policy, Management and Evaluation, University of Toronto, Toronto, Ontario, Canada; 18 London Health Sciences Centre-University Campus, Western University, London, Ontario, Canada; 19 1st Department of Medicine, Semmelweis University, Budapest, Hungary; 20 Crohn’s and Colitis Canada, Toronto, Ontario, Canada; 21 CHEO Inflammatory Bowel Disease Centre and Department of Pediatrics, University of Ottawa, Ottawa, Ontario, Canada; 22 Department of Internal Medicine, King Abdulaziz University Hospital, Jeddah, Saudi Arabia; 23 The Ottawa Hospital IBD Centre, Department of Medicine, University of Ottawa, Ottawa, Ontario, Canada; 24 Mount Sinai Hospital Inflammatory Bowel Disease Centre, University of Toronto, Toronto, Ontario, Canada; 25 Division of Gastroenterology and Hepatology, Department of Medicine, University of Calgary, Calgary, Alberta, Canada; 26 Division of Gastroenterology and Hepatology, Mount Sinai Hospital, University of Toronto, Toronto, Ontario, Canada

**Keywords:** Coronavirus, Crohn’s disease, SARS-CoV-2, Ulcerative colitis

## Abstract

Persons with inflammatory bowel disease (IBD) make up more than 0.75% of the Canadian population in 2021. Early in the COVID-19 pandemic, individuals with IBD, particularly those on immunosuppressive therapies, were concerned that their health status may place them at higher risk of contracting COVID-19 or experiencing more severe disease course if infected with SARS-CoV-2. In response, Crohn’s and Colitis Canada developed the COVID-19 and IBD Taskforce in March 2020 to rapidly synthesize the evolving knowledge of COVID-19 as relevant to Canadians with IBD. The Taskforce communicated expert information directly to the Canadian IBD community through online tools and a webinar series. In order to understand the full impact of COVID-19 on the IBD community, Crohn’s and Colitis Canada commissioned a policy report that was informed through a systematic literature review and synthesized across working groups along the following domains: Epidemiology, Children and Expectant Mothers with IBD, Seniors with IBD, Mental Health, Risk Factors and Medications, Vaccines, and Healthcare Delivery during the Pandemic and the Future Model of IBD Care. This report from Canadian physicians, researchers, and IBD community representatives highlights the physical, mental, and health systems impact of COVID-19 on the entire spectrum of the IBD community, including children, adolescents, adults, seniors, and pregnant people with IBD. This executive summary provides an overview of the crucial information from each of the chapters of the policy report, supplemented with additional information made available through Crohn’s and Colitis Canada’s webinar-based knowledge translation platform.

**KEY POINTS**

1. In response to the COVID-19 pandemic, Crohn’s and Colitis Canada’s COVID-19 and IBD taskforce synthesized relevant knowledge on a regular basis and communicated with the IBD community in real-time through expert-generated online tools and frequent webinars for a public audience.
2. The transmissibility of SARS-CoV-2 for those with IBD follows epidemiologic trends within the general population; most people with IBD are not at increased risk of contracting COVID-19 or having severe COVID-19 compared to the general population.
3. Children with IBD have milder COVID-19 disease course and are less likely to require hospitalization compared to adults with IBD, which is consistent with the observations on the natural history of COVID-19 across the age spectrum in the general population.
4. In the general population, pregnancy is associated with more severe COVID-19 or birth complications; however, the impact of COVID-19 in pregnant people with IBD is not known.
5. Seniors have the highest risk of severe COVID-19 in the general population and among those with IBD.
6. Persons with IBD who are flaring and require a high dose of corticosteroids are at higher risk of acquiring or experiencing severe complications from COVID-19.
7. Biologic maintenance therapies for IBD are not associated with a higher risk of acquiring COVID-19 or having a more serious course following infection from SARS-CoV-2.
8. As a result of the COVID-19 pandemic, an increase in distress and worsening mental health has been demonstrated in children, adolescents, adults, seniors, and pregnant people with and without IBD.
9. Real-world evidence suggests that vaccines for COVID-19 are safe and elicit robust immune responses to SARS-CoV-2 infection following two doses of vaccine in individuals with IBD, although immunity may be less robust following a single dose of vaccine among those receiving anti-TNF therapy or conventional immunosuppressants.
10. COVID-19 has necessitated alternate models of delivering care to those with IBD, including virtual care and remote monitoring of disease activity, which will influence health care delivery models beyond the SARS-CoV-2 pandemic.

## INTRODUCTION

Inflammatory bowel disease (IBD) affects more than 0.75% of the Canadian population in 2021 (1–5). At the onset of the pandemic, individuals with chronic immune-mediated inflammatory diseases, such as IBD, and those on immunocompromising therapies were identified as potential vulnerable populations with an unknown risk of SARS-CoV-2 infection (6). In parallel, Canadian health care providers initially lacked evidence-based guidance, leading to a potential risk of heterogeneous communication and management of IBD during the pandemic.

In response, Crohn’s and Colitis Canada (CCC) developed a national representative taskforce made up of physicians (adult and pediatric gastroenterologists, infectious disease specialists), scientists, nurses, and IBD community representatives. In March 2020, CCC formed the COVID-19 and IBD Taskforce (https://crohnsandcolitis.ca/About-Crohn-s-Colitis/COVID-19-and-IBD/Task-Force) to synthesize the literature on COVID-19 and make recommendations relevant to the IBD community in Canada. Knowledge from the Taskforce was communicated to the IBD Community through CCC’s website and a regular webinar series moderated by the co-chairs of the Taskforce, featuring invited panelists who addressed specific topics on COVID-19 and IBD. As of May 2021, CCC’s webpage on COVID-19 has been viewed nearly half a million times, and 25 webinars have been produced on COVID-19 and IBD that have been viewed nearly 130,000 times.

CCC commissioned a policy report on COVID-19’s impact on the IBD community at the 1-year anniversary of the World Health Organization’s declaration of a global pandemic. This executive summary explains the sections of the policy report: Epidemiology, Children and Expectant Mothers with IBD, Seniors with IBD, Mental Health, Risk Factors and Medications, Vaccines, and Healthcare Delivery during the Pandemic and the Future Model of IBD Care. Integrated into each section is our knowledge translation strategy to communicate the impact of COVID-19 on the IBD community. This executive summary provides an overview of the crucial information from each of the chapters of the policy report, supplemented with additional information made available through CCC’s webinar-based knowledge translation platform.

## METHODS

A steering committee (EB, CB, AB, JJ, GK, MEK, KL, SM, JW, LT) was established to evaluate the impact of COVID-19 on the IBD community living in Canada. The Crohn’s and Colitis Canada’s COVID-19 and Inflammatory Bowel Disease 2021 Impact Report was stratified into working groups corresponding with the following sections in this report: epidemiology (SC, EB, CB, AB, JJ, GK, RK, MEK, JW); Children and Expectant Mothers (EB, MC, RG, AG, DM, CS); Seniors (CB, HS, SM, GN, GK); Mental Health (LG, SF, JJ, CB); Risk Factors and Medications (LT, CB, PL, SM, RP); Vaccines (SM, JG, DG, AG, MEK, RP, CS); and Healthcare Delivery during the Pandemic and the Future Model of IBD Care (JJ, EB, CB, JM, SM, GN, AB).

A systematic review of the literature established the evidence used by the working groups to create the sections in this report. We searched MEDLINE, EMBASE, PsycINFO, CINAHL, Cochrane Library, the Cochrane COVID-19 Study Register, the World Health Organization’s (WHO) COVID-19 database (https://search.bvsalud.org/global-literature-on-novel-coronavirus-2019-ncov/) from 2019 to February 4, 2021 with an update on March 24, 2021 to identify studies describing the impact of the COVID-19 pandemic on people living with IBD. The WHO database includes preprints of all COVID-19 publications from medRxiv and bioRxiv. The search strategy included search terms for IBD (including Crohn’s disease and ulcerative colitis) and COVID-19 and is outlined in full in Supplementary Table 1. Our search was left intentionally broad to ensure all relevant articles were identified. Additional studies published since the end of the literature search were identified through active surveillance of the published literature.

The search of each database was limited according to the available filters in each database. CINAHL and the Cochrane COVID-19 Study Register were searched from February 2021. This updated search included all articles indexed in MEDLINE, EMBASE, and PsycINFO from January 1, 2021. All articles were identified from the Cochrane Library and WHO database. For database searches with overlapping search dates, duplicate references were removed manually. Any articles included in the literature search published prior to 2019 were also excluded.

We included any study reporting on the impact of the COVID-19 pandemic on persons with IBD, including the following: The epidemiology of COVID-19 in persons with IBD; specific impacts of the pandemic on children or seniors, including their families/caregivers; pregnant people with IBD and/or their newborns; changes in health care delivery, including limited access to endoscopy and elective surgery; mental health; vaccines; and quality of life. We included both primary studies and review articles, as well as any clinical practice guidelines, recommendations, or opinion pieces about the care of individuals with IBD during the pandemic. We excluded study protocols, case series of <10 participants, basic or translational science studies, and studies describing the impact of COVID-19 on the gastrointestinal system. No restrictions were placed on the language of publication, and we included studies published in abstract form.

Abstracts of studies identified by the literature search were independently screened by two individuals (JGH, PT, or MSM). The full-text of relevant abstracts was then independently reviewed by two individuals (JGH, PT, or MSM). Conflicts at both stages were reviewed by a third reviewer (MEK). Covidence was used to facilitate the review of abstracts and full texts (7).

**Figure 1.**
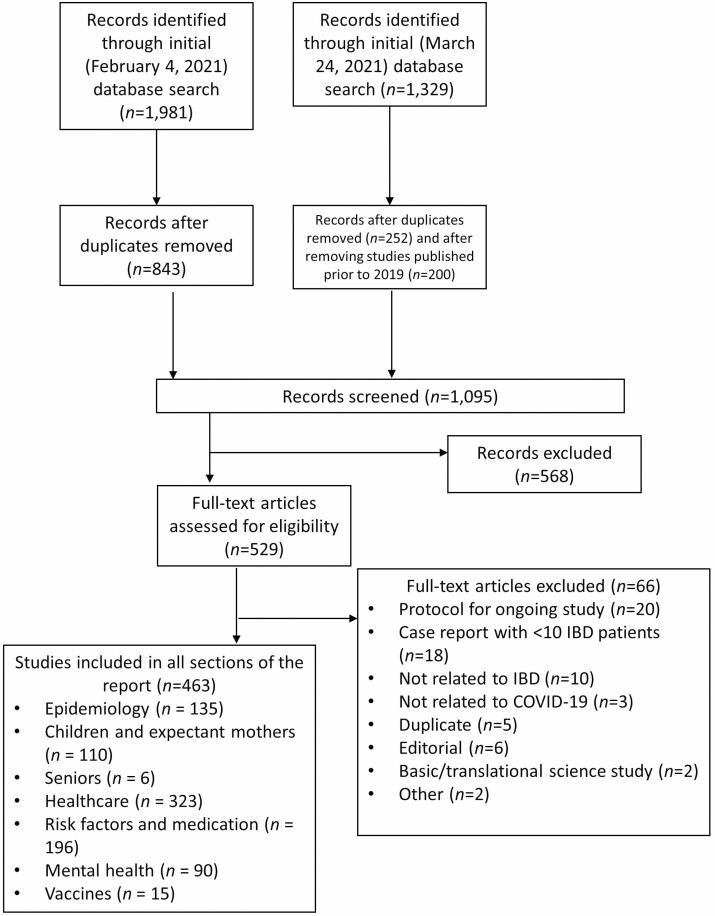
PRISMA flow diagram.

A total of 1,981 references were identified in the initial search (February 4, 2021) and 1,329 additional references were identified in the update (March 24, 2021); 1,105 records remained after removing duplicates (Figure 1). The full texts of 529 studies were assessed for eligibility and 463 were included. Studies pertinent to each chapter of the report were tagged based on the topic or theme of the paper, then distributed to authors of that chapter.

### The Impact of COVID-19 on IBD: Report Summary

#### Epidemiology

SARS-CoV-2 became a global pandemic because of the high transmissibility of the virus, the severe morbidity and mortality of COVID-19 that overwhelmed health care systems, and the novelty of the virus leaving the global population susceptible to infection (8). Without public health interventions to control the spread of SARS-CoV-2, the effective reproductive number (*R*_*t*_) of the virus led to exponential spread across the globe. Public health measures from lockdowns to mask mandates reined in the periodic waves of infections across Canada, serving as a barrier against the virus until vaccines were available and distributed.

Reassuringly, evidence that emerged over the last year consistently demonstrated that the risk of contracting SARS-CoV-2 or experiencing severe COVID-19 if acquired was similar in those with Crohn’s disease and ulcerative colitis as compared to the general population; for example, seniors with IBD were at highest risk for hospitalization and death following infection with SARS-CoV-2 regardless of whether they had IBD or not (9–11). However, most studies do not account for potential differences in adherence to public health guidelines among people with IBD and the general population. Specific to the IBD community, those with actively flaring disease, particularly those requiring high dose corticosteroids (>20 mg per day), were at risk of the worst outcomes from COVID-19 (12–14).

The epidemiology of COVID-19 in those with IBD were regularly communicated to the IBD community through the Crohn’s and Colitis Canada’s COVID-19 and IBD webinar series, whereby the co-moderator (GK) presented on the current global, Canadian, and IBD-specific epidemiology of COVID-19. On March 19, 2020 (the first webinar), 782 Canadians were diagnosed with COVID-19; globally, 21 people with IBD who had contracted COVID-19 were reported in the Surveillance Epidemiology of Coronavirus Under Research Exclusion (SECURE-IBD) registry (15). One year later (March 18, 2021, after 23 webinars) nearly 1 million Canadians and 6,000 individuals with IBD, worldwide, had contracted COVID-19 (16).

#### Children and Expectant Mothers with IBD

The Taskforce emphasized several special topics, including the impact of COVID-19 on children and pregnant people with IBD. From inception, the recommendations were age-based, with an emphasis on the mild course experienced by most children with COVID-19, even when immunocompromised due to their IBD medications (17). Despite the typically mild disease course, the Taskforce deemed it important to address the concerns of parents with respect to reducing transmission, especially when in-person schooling resumed. Back-to-school recommendations were incorporated into the recommendations, and a unique webpage (https://crohnsandcolitis.ca/About-Crohn-s-Colitis/COVID-19-and-IBD/Back-to-School) was created to address the concerns of parents and teachers. In addition to recommendations for at-risk individuals, the Taskforce made recommendations for family members of at-risk children (such as the siblings of people on systemic corticosteroids) to emphasize the importance of shielding the individual from COVID-19 while enabling siblings to attend in-person school and parents to return to work (18). Pregnant individuals are at greater risk of a more severe COVID-19 disease course or birth complications; however, evidence on the additional impact of IBD on the likelihood of experiencing more severe COVID-19 in pregnancy is lacking.

All the webinars included pediatric health care provider representation. Additionally, multiple webinars were focused on educating parents of children with IBD (e.g., Families and Children with IBD: May 7, 2020; https://crohnsandcolitis.ca/About-Crohn-s-Colitis/COVID-19-and-IBD/COVID-19-Webinars/COVID-19-Families-and-Children-with-IBD) and people who were pregnant (e.g., Pregnancy and Newborns: March 26, 2020; https://crohnsandcolitis.ca/About-Crohn-s-Colitis/COVID-19-and-IBD/COVID-19-Webinars/COVID-19-and-IBD-Medications). Subsequent webinars also focused on back-to-school recommendations (e.g., Returning to School: September 10, 2020; https://crohnsandcolitis.ca/About-Crohn-s-Colitis/COVID-19-and-IBD/COVID-19-Webinars/COVID-19-Updates-and-Returning-to-School).

#### Seniors With IBD

Seniors with IBD represent a highly prevalent population that must contend with managing IBD in the context of age-related comorbidities (19). The COVID-19 pandemic imposed tremendous stress on the elderly IBD population, as seniors had the highest risk of acquiring COVID-19 or experiencing severe COVID-19 if contracted (defined as hospitalization, ICU admission, or death). However, emerging evidence suggests that seniors with IBD are not at increased risk of acquiring COVID-19 or experiencing worse outcomes from COVID-19 as compared to seniors in the general population (11,20). Nonetheless, due to the higher risk status of the senior population, a number of health care adaptations were instituted to reduce risk of exposure of seniors with IBD to SARS-CoV-2, such as increased emphasis on telemedicine. While the elderly may have less robust immune responses to vaccines, experiences from other vaccination programs, especially for influenza, have shown that vaccinating the elderly reduces both morbidity and mortality and, in turn, health care resources (21). Thus, messaging from Crohn’s and Colitis Canada through the webinar series has consistently supported recommendations to protect seniors with IBD from exposure to SARS-CoV-2 and to advocate for high rates of vaccine uptake.

#### Risk Factors and Medications

In March 2020, as Canada entered the first societal lockdown, individuals with IBD and their health care providers were making decisions on health care management without evidence regarding the impact of COVID-19 on IBD. Initially, guidance was based on expert consensus opinion (6). However, by July 2020, the first 500 cases from the SECURE-IBD registry indicated that the primary risk factor for a poor outcome of COVID-19 was age. Maintenance therapies, such as anti-TNFs, were not associated worse outcomes; though flaring individuals who required high dose corticosteroids were more likely to be hospitalized or die from COVID-19 (12). These data were substantiated in later studies from the SECURE-IBD registry as well as from different study populations (11,14,20).

Consequently, the COVID-19 and IBD Taskforce consistently communicated a message to the IBD community not to discontinue effective therapy because cessation of treatment in fear of COVID-19 may lead to a flare, which is associated with an increased risk of worse outcomes from COVID-19 (13). Several webinars (e.g., IBD Medications and COVID-19 Risk: June 18, 2020; https://crohnsandcolitis.ca/About-Crohn-s-Colitis/COVID-19-and-IBD/COVID-19-Webinars/IBD-Medications-and-COVID-19-Risk) were dedicated to communicate efficacy and safety of IBD medications throughout the pandemic.

#### Mental Health and Quality of Life

Early in the COVID-19 pandemic, community anxiety levels, distress, and depressive symptoms were elevated, which was recognized as a normal response to a highly stressful situation. Individuals with IBD and health care providers were concerned that the heightened stress as a result of the pandemic would lead to not just mental health disorders but also exacerbation of disease activity (22). During our webinar series (e.g., Mental Health and Wellness: May 28, 2020; https://crohnsandcolitis.ca/About-Crohn-s-Colitis/COVID-19-and-IBD/COVID-19-Webinars/COVID-19-Mental-Health-and-Wellness), information was provided on common psychologic reactions to the pandemic, including heightened anxiety, fear, and/or depression, noting reported increases in alcohol and cannabis use. Several factors have contributed to mental health difficulties during the pandemic, including uncertainty regarding risks of contracting COVID-19 and challenges of public health restrictions, social isolation, and financial stressors. Webinars and online communication provided guidance on managing the stress of the pandemic, including Relaxation and breathing exercises, mindfulness meditation, exercising at home, keeping a regular sleep routine, maintaining connection with friends and family, limiting COVID-19 information overload, and ensuring that IBD care and follow-up is maintained.

#### COVID-19 Vaccines and IBD

Vaccines to SARS-CoV-2 hold the promise of protecting individuals who may be at higher risk, such as those who are immunocompromised (23). However, the mRNA vaccines (produced by Pfizer and BioNTech, and Moderna) and nonreplicating viral vector (produced by AstraZeneca and Janssen) currently approved by Health Canada, were not originally studied in persons with immune-mediated diseases or those on immunosuppressant medications, including those with IBD (24–26). However, emerging data have demonstrated that the COVID-19 vaccines are safe and effective in IBD patients. The CLARITY IBD study assessed vaccine antibody response in those with IBD on biologics. The seroconversion rate after the first dose of vaccine was lower among individuals on infliximab as compared to those on vedolizumab, particularly in people on concomitant immunomodulators (27). However, seroconversion was robust following the second dose of the vaccine and after the first dose among those who had recovered from a prior infection to COVID-19 (27). Furthermore, a national US cohort study of nearly 15,000 people with IBD reported 80.4% vaccine effectiveness among those who were at least seven days from their second dose of mRNA vaccine (28). Early studies have further shown positive benefits and negligible risk of COVID-19 vaccination among adolescents aged 12 to 15 years and among pregnant people (29). Canadian (30), European (31), and international (32) organizations recommend that all individuals with IBD be vaccinated against SARS-CoV-2 at the earliest opportunity, irrespective of vaccine type, disease status or treatment, and without interruption of scheduled therapy.

Crohn’s and Colitis Canada has strongly advocated for persons with IBD to receive a scheduled second dose of vaccine three to four weeks following the first dose to boost immunity—in line with the schedule followed in the trials—rather than extending the interval for the second dose. Education of vaccine safety and efficacy in the IBD population occurred regularly in our webinar series and evolved monthly as real-world evidence was published regarding safety and efficacy the IBD population. The webinars (e.g., Vaccine Updates and Recommendations: April 24, 2021; https://crohnsandcolitis.ca/About-Crohn-s-Colitis/COVID-19-and-IBD/COVID-19-Webinars/Vaccine-Updates-and-Recommendations) focused on providing the most up-to-date information with the goal of optimizing vaccine uptake by the IBD community.

#### Health Care Delivery During the Pandemic and the Future Model of IBD Care

The COVID-19 pandemic dramatically shifted access to and delivery of care for Canadians living with IBD. During the first wave of the pandemic, in the spring of 2020, health care systems entirely redesigned ambulatory, diagnostic, and hospital care. The majority of health care delivery shifted to virtual telemedicine (phone or videoconference), whereas in-person health care delivery was transformed to adopt strict physical distancing, hygiene, wearing face masks, and personal protective equipment. These measures conserved health system capacity and reduced potential transmission of SARS-CoV-2. The rapid and widespread innovation of health care delivery from the pre-pandemic era was not without precedent; (33) Crohn’s and Colitis Canada had already developed the Promoting Access and Care through Centers of Excellence (PACE) Telemedicine Program in Ontario (34). Moreover, during the pandemic, studies of people with IBD and health care providers identified increased satisfaction, improved outcomes, and greater efficiency with the health care innovations (e.g., Telemedicine) such that the future of IBD care will most likely comprise hybrid models of in-person and virtual health care delivery (35).

The COVID-19 and IBD webinar series highlighted the adoption of health care innovation (e.g., Telemedicine in the Time of The Pandemic: April 30, 2020; https://crohnsandcolitis.ca/About-Crohn-s-Colitis/COVID-19-and-IBD/COVID-19-Webinars/COVID-19-Updates-Telemedicine-in-the-Time-of-the) and the future delivery of care (e.g., IBD Clinic of the Future: June 11, 2020). The key message from these webinars and emerging studies was that the lessons learned during the pandemic will influence models of delivering IBD care well beyond the SARS-CoV-2 pandemic.

## CONCLUSION

Crohn’s and Colitis Canada’s COVID-19 and IBD Taskforce produced online tools and webinars that served to rapidly synthesize and communicate scientific knowledge on COVID-19’s impact on the IBD community. During the first year of the pandemic, clinical evidence indicated that most individuals with IBD followed similar epidemiologic and clinical patterns of the general population following infection with SARS-CoV-2. Across the spectrum of children, adolescents, adults, seniors, and pregnant people with IBD who maintained remission with their medical therapies, the risk of transmission and disease severity paralleled the general population. In contrast, individuals with IBD with moderate to severe active inflammation and who required a high dose of corticosteroids (>20 mg per day) were at higher risk of severe complications from COVID-19. Increases in distress and mental health disorders, including anxiety and depression, were prevalent across the age span, creating a significant burden on persons with IBD. The rapidly evolving data on vaccines to SARS-CoV-2 revealed that administering two doses of vaccine was safe and elicited a robust immune response against SARS-CoV-2 infection in those with IBD. Finally, the paradigm shifting changes to the delivery of care in order to prevent transmission of SARS-CoV-2, such as widespread virtual care and remote monitoring of disease activity, led to innovations in health care delivery models that will persist beyond the SARS-CoV-2 pandemic.

## Supplementary Material

gwab027_suppl_Supplementary_Table
