# Supplementary material for: Crohn’s and Colitis Canada’s 2021 Impact of COVID-19 and Inflammatory Bowel Disease in Canada: Executive Summary
Source: J Can Assoc Gastroenterol. 2021 Nov 5;4(Suppl 2):S1–9. doi: 10.1093/jcag/gwab027 (PMC8570424; doi:10.1093/jcag/gwab027)
Supplement: gwab027_suppl_Supplementary_Table [file gwab027_suppl_supplementary_table.docx]

**SUPPLEMENTAL FILE**

**EMBASE (Ovid)**

1. exp Coronavirus infection/
2. exp Coronavirinae/
3. exp coronaviridae/
4. exp coronaviridae infection/
5. (2019 nCoV or 2019nCoV or 2019-novel CoV).ti,ab,kw.
6. COVID.af.
7. (COVID19 or "COVID-19" or "COVID 19").ti,ab,kw.
8. (nCov 2019 or nCov 19).ti,ab,kw.
9. ("SARS-CoV-2" or "SARS-CoV2" or SARSCoV2 or "SARSCoV-2" or SARS2).af.
10. ("SARS coronavirus 2" or "SARS-like coronavirus" or "Severe Acute Respiratory Syndrome Coronavirus-2" or "Severe Acute Respiratory Syndrome Coronavirus 2").af.
11. (("severe acute respiratory" or pneumonia*) adj5 (coronavir* or COVID*)).af.
12. (corona vir* or coronavir* or neocorona vir* or neocoronavir*).ti,ab,kw.
13. ((wuhan or Hubei or Hunan) and (severe acute respiratory or pneumonia*) and outbreak*).mp.
14. ((wuhan or hubei or Hunan) and coronavir*).mp.
15. or/1-14
16. Inflammatory bowel disease/
17. exp Crohn disease/
18. exp ulcerative colitis/
19. proctitis/
20. ileitis/
21. pancolitis/
22. proctocolitis/
23. ("Inflammatory bowel disease*" or ulcerative colitis or Crohn* or IBD or ileocolitis or ileitis or pancolitis or proctitis).af.
24. or/16-23
25. 15 and 24
26. limit 25 to yr="2019 -Current"

**WHO COVID-19 Database** (https://search.bvsalud.org/global-literature-on-novel-coronavirus-2019-ncov/)

tw:("Inflammatory bowel disease" OR "inflammatory bowel diseases" OR "crohn disease" OR "crohn's disease" OR "ulcerative colitis" OR ibd OR ileocolitis OR ileitis OR pancolitis OR proctitis)

**Cochrane Library (Wiley)**

| ID | Search |
| --- | --- |
| #1 | MeSH descriptor: [SARS-CoV-2] explode all trees |
| #2 | MeSH descriptor: [Coronavirus] explode all trees |
| #3 | MeSH descriptor: [Severe Acute Respiratory Syndrome] this term only |
| #4 | MeSH descriptor: [COVID-19] this term only |
| #5 | MeSH descriptor: [Coronaviridae] explode all trees |
| #6 | MeSH descriptor: [Coronavirus Infections] this term only |
| #7 | ("2019 nCoV"):ti,ab,kw OR ("2019nCoV"):ti,ab,kw OR ("2019-novel CoV"):ti,ab,kw |
| #8 | (COVID):ti,ab,kw OR (COVID19):ti,ab,kw OR ("COVID-19"):ti,ab,kw |
| #9 | ("nCov 2019"):ti,ab,kw OR ("nCov 19"):ti,ab,kw |
| #10 | ("SARS-CoV-2"):ti,ab,kw OR ("SARS-CoV2"):ti,ab,kw OR (SARSCoV2):ti,ab,kw OR ("SARSCoV-2"):ti,ab,kw OR (SARS2):ti,ab,kw |
| #11 | ("SARS coronavirus 2"):ti,ab,kw OR ("SARS-like coronavirus"):ti,ab,kw OR ("Severe acute respiratory syndrome coronavirus-2"):ti,ab,kw OR ("Severe acute respiratory syndrome coronavirus 2"):ti,ab,kw |
| #12 | (("severe acute respiratory"):ti,ab,kw OR (pneumonia*):ti,ab,kw) AND ((coronavir*):ti,ab,kw OR (COVID*):ti,ab,kw) |
| #13 | ("corona vir*"):ti,ab,kw OR (coronavir*):ti,ab,kw OR ("neocorona vir*"):ti,ab,kw OR (neocoronavir*):ti,ab,kw |
| #14 | ((wuhan):ti,ab,kw OR (Hubei):ti,ab,kw OR (Hunan):ti,ab,kw) AND (("severe acute respiratory"):ti,ab,kw OR (pneumonia*):ti,ab,kw) AND (outbreak*):ti,ab,kw |
| #15 | ((wuhan):ti,ab,kw OR (Hubei):ti,ab,kw OR (Hunan):ti,ab,kw) AND ((coronavir*):ti,ab,kw OR (COVID*):ti,ab,kw) |
| #16 | {OR #1-#15} |
| #17 | MeSH descriptor: [Inflammatory Bowel Diseases] explode all trees |
| #18 | MeSH descriptor: [Crohn Disease] explode all trees |
| #19 | MeSH descriptor: [Colitis, Ulcerative] explode all trees |
| #20 | MeSH descriptor: [Proctitis] this term only |
| #21 | MeSH descriptor: [Ileitis] explode all trees |
| #22 | MeSH descriptor: [Proctocolitis] this term only |
| #23 | ("inflammatory bowel disease*"):ti,ab,kw OR ("ulcerative colitis"):ti,ab,kw OR (Crohn*):ti,ab,kw OR (IBD):ti,ab,kw |
| #24 | (ileocolitis):ti,ab,kw OR (ileitis):ti,ab,kw OR (pancolitis):ti,ab,kw OR (proctitis):ti,ab,kw |
| #25 | {OR #17-#24} |
| #26 | #16 AND #25 |

**Cochrane COVID-19 Study Register** (covid-19.cochrane.org)

“Inflammatory bowel disease*” or ulcerative colitis OR Crohn* or IBD or ileocolitis or ileitis or pancolitis or proctitis

**CINAHL (EBSCO)**

| Search ID | Search Terms |
| --- | --- |
| S20 | S12 AND S18 |
| S19 | S12 AND S18 |
| S18 | S13 OR S14 OR S15 OR S16 OR S17 |
| S17 | TX ("Inflammatory bowel disease*" or ulcerative colitis or Crohn* or IBD or ileocolitis or ileitis or pancolitis or proctitis) |
| S16 | (MH "Ileitis+") |
| S15 | (MH "Colitis, Ulcerative") |
| S14 | (MH "Crohn Disease") |
| S13 | (MH "Inflammatory Bowel Diseases+") |
| S12 | S1 OR S2 OR S3 OR S4 OR S5 OR S6 OR S7 OR S8 OR S9 OR S10 OR S11 |
| S11 | TX ((wuhan or hubei or Hunan) and coronavir*) |
| S10 | TX ((wuhan or Hubei or Hunan) and (severe acute respiratory or pneumonia*) and outbreak*) |
| S9 | TX (corona vir* or coronavir* or neocorona vir* or neocoronavir*) |
| S8 | TX ( "severe acute respiratory" OR pneumonia* ) AND TX ( coronavir* OR COVID* ) |
| S7 | TX ("SARS coronavirus 2" or "SARS-like coronavirus" or "Severe Acute Respiratory Syndrome Coronavirus-2" or "Severe Acute Respiratory Syndrome Coronavirus 2") |
| S6 | TX ("SARS-CoV-2" or "SARS-CoV2" or SARSCoV2 or "SARSCoV-2" or SARS2) |
| S5 | TX nCov 2019 or nCov 19 |
| S4 | TX (COVID19 or "COVID-19" or "COVID 19") |
| S3 | TX COVID |
| S2 | TX (2019 nCoV or 2019nCoV or 2019-novel CoV) |
| S1 | (MH "Coronavirus+") OR (MH "Coronavirus Infections+") OR (MH "COVID-19") |

**MEDLINE (Ovid)**

1. exp Coronavirus/
2. exp Coronavirus Infections/
3. exp coronaviridae/
4. exp coronaviridae infections/
5. (2019 nCoV or 2019nCoV or 2019-novel CoV).ti,ab,kw.
6. COVID.af.
7. (COVID19 or "COVID-19" or "COVID-19").ti,ab,kw.
8. (nCov 2019 or nCov 19).ti,ab,kw.
9. ("SARS-CoV-2" or "SARS-CoV2" or SARSCoV2 or "SARSCoV-2" or SARS2).af.
10. ("SARS coronavirus 2" or "SARS-like coronavirus" or "Severe Acute Respiratory Syndrome Coronavirus-2" or "Severe Acute Respiratory Syndrome Coronavirus 2").af.
11. (("severe acute respiratory" or pneumonia*) adj5 (coronavir* or COVID*)).af.
12. (corona vir* or coronoavir* or neocorona vir* or neocoronavir*).ti,ab,kw.
13. ((wuhan or hubei or Hunan) and (severe acute respiratory or pneumonia*) and outbreak*).mp.
14. ((wuhan or hubei or Hunan) and coronavir*).mp.
15. or/1-14 [Set 1: SARS-CoV-2]
16. exp Inflammatory Bowel Diseases/
17. exp Colitis, Ulcerative/
18. exp Crohn Disease/
19. exp Proctitis/
20. exp Enteritis/
21. ("Inflammatory bowel disease*" or ulcerative colitis or Crohn* or IBD or ileocolitis or ileitis or pancolitis or proctitis).af.
22. or/16-21 [Set 2: IBD]
23. 15 and 22
24. limit 23 to yr="2019 -Current"

**PsycINFO (Ovid)**

1. exp coronavirus/
2. (2019 nCoV or 2019nCoV or 2019-novel CoV).mp.
3. COVID.af.
4. (COVID19 or "COVID-19" or "COVID 19").mp.
5. (nCov 2019 or nCov 19).mp.
6. ("SARS-CoV-2" or "SARS-CoV2" or SARSCoV2 or "SARSCoV-2" or SARS2).af.
7. ("SARS coronavirus 2" or "SARS-like coronavirus" or "Severe Acute Respiratory Syndrome Coronavirus-2" or "Severe Acute Respiratory Syndrome Coronavirus 2").af.
8. (("severe acute respiratory" or pneumonia*) adj5 (coronavir* or COVID*)).af.
9. (corona vir* or coronavir* or neocorona vir* or neocoronavir*).mp.
10. ((wuhan or Hubei or Hunan) and (severe acute respiratory or pneumonia*) and outbreak*).mp.
11. ((wuhan or hubei or Hunan) and coronavir*).mp.
12. or/1-11 [Set 1: SARS-CoV-2]
13. exp colon disorders/
14. exp ulcerative colitis/ or exp colitis/
15. ("Inflammatory bowel disease*" or ulcerative colitis or Crohn* or IBD or ileocolitis or ileitis or pancolitis or proctitis).af.
16. or/13-15
17. 12 and 16
